# How green marathons are associated with pro-sustainable behavior: the chain-mediating roles of environmental empathy and self-sustainable identity

**DOI:** 10.3389/fpsyg.2026.1731205

**Published:** 2026-04-20

**Authors:** Kui Dong, Yang Wang, Shengtao Wang, Jun Tong, Renfa Zhang, Keran Li, Yu Cheng, Yang Lan

**Affiliations:** 1 Department of Physical Education, Kunming Medical University, Kunming, Yunnan, China; 2 School of International Education, Kunming Medical University, Kunming, Yunnan, China

**Keywords:** chain mediation, cognitive-affective-conative (CAC) theory, environmental awareness, environmental empathy, Green Marathon, pro-sustainability behavior, self-sustainable identity

## Abstract

The global rise of green marathons has sparked interest in their potential to be associated with pro-sustainable behaviors. However, the mechanisms underlying this process are not well understood. Grounded in the cognitive-affective-conative (CAC) theory, this study proposes a model wherein three dimensions of environmental awareness (environmental value integration, green behavior belongingness, and collective environmental efficacy) be associated with pro-sustainable behavior tendencies through the chain mediation of environmental empathy and self-sustainable identity. Data from 651 participants were analyzed using structural equation modeling (SEM). The results showed that: (1) All three dimensions of environmental awareness positively influenced pro-sustainable behavior tendencies. (2) Environmental empathy and self-sustainable identity sequentially mediated the relationship between environmental value integration and pro-sustainable behavior tendencies. (3) A similar chain mediation effect was found for green behavior belongingness. (4) Collective environmental efficacy also influenced pro-sustainable behavior tendencies through the sequential pathway of environmental empathy and self-sustainable identity. This study provides a novel theoretical framework for understanding how green marathons be associated with sustainable behavior, highlighting the critical roles of emotional and identity pathways. The findings offer practical insights for organizers and policymakers, emphasizing the need to cultivate environmental empathy and self-sustainable identity in the design of green events.

## 1 Introduction

Against the backdrop of rapid industrialization, uncontrolled industrial production in modern societies has led to excessive resource consumption and severe environmental pollution, placing the world’s fragile ecosystems under unprecedented pressure (Hoose and Kripka, 2021). In this process, environmental issues such as air, water, and soil pollution continue to intensify, directly impacting human health and well-being (Mohamad et al., 2022). Fortunately, societal awareness of environmental protection is gradually rising. Groups including environmental activists, organizations, individual advocates, and vegetarians are expanding rapidly beyond their original circles, forming a new wave of environmental protection trends (Perez et al., 2015; Giugni and Grasso, 2015; Pei et al., 2023; Chen et al., 2024). In the face of a deteriorating ecological environment, the question of how to stimulate individual pro-sustainable behaviors to address the global ecological crisis has become increasingly urgent.

As the sports industry evolves into a global blue ocean market and attracts growing capital investment, the environmental issues associated with it are often overlooked (Watanabe et al., 2023). Taking the marathon as an example—one of the world’s most popular sporting events—these events typically involve large numbers of participants, spectators, volunteers, and organizers. Issues such as transportation demands, waste accumulation along the course, and discarded packaging from beverages and energy foods impose varying levels of burden on the environment (Marr and Ely, 2010). Waste and carbon emissions generated by the millions of marathons held worldwide each year are gradually drawing attention from environmental organizations (Guo and Fu, 2019). Therefore, how to mitigate negative environmental impacts while maintaining the scale and influence of such events has become a critical challenge for the sports industry. In this context, the “Green Marathon,” as an innovative event format integrating health, environmental protection, and social engagement, has successfully attracted numerous environmentalists and the general public by promoting low-carbon travel and a green, healthy lifestyle (Triantafyllidis and Kaplanidou, 2021). The Green Marathon is not only a sporting event but also a new channel for environmental communication, embodying the core concept of “sports as the framework, green as the spirit” (Triantafyllidis and Kaplanidou, 2022). Under the theme of “Going Green,” it raises participants’ awareness of environmental issues and encourages the public to consciously adopt more eco-friendly behaviors in their daily lives. However, despite the growing popularity of green marathons worldwide, there remains a significant research gap regarding how these events can enhance participants’ environmental awareness to promote pro-sustainable behaviors.

Based on this, the present study adopts an interdisciplinary perspective integrating environmental behavior and social psychology. Grounded in the cognitive–affective–conative (CAC) theory as the primary framework, and drawing on communication and community perspectives only to contextualize green-event participation, we examine how the three-dimensional environmental awareness fostered in Green Marathon activities is associated with participants’ pro-sustainable behavior tendencies through the chain-mediating roles of environmental empathy and self-sustainable identity. By applying structural equation modeling (SEM) to psychological survey data from 651 Green Marathon participants, this study tests the proposed mechanism and clarifies the roles of emotional engagement and identity-based volitional tendency in translating ecological awareness into pro-sustainable behavioral tendencies. The findings provide a process-oriented account of sustainable behavior formation in a green-event setting and offer practical implications for the design and governance of future green events.

This study makes three contributions to the pro-environmental behavior literature. First, beyond prior work that typically examines either emotion-based (e.g., empathy) or identity-based predictors in isolation, we test a sequential mechanism in which environmental awareness fosters environmental empathy, which in turn promotes a self-sustainable identity and ultimately pro-sustainable behavior. This chain highlights empathy-to-identity as an ordered psychological process rather than parallel predictors. Second, we advance event-based sustainability research by demonstrating how a Green Marathon—an intensive, collective, and goal-directed event setting—can function as a salient context for identity formation, thereby extending identity formation arguments beyond stable traits to situationally activated self-definitions. Third, we refine cognitive–affective–conative (CAC) theory in green event contexts by operationalizing the conative component as an identity-based volitional tendency (self-sustainable identity) and examining whether cognition translates into behavior primarily through affective engagement and subsequent conative activation. Together, these contributions clarify what is unique about Green Marathon contexts and why the proposed chain offers incremental explanatory value over existing models.

Green marathons represent a psychologically distinctive sustainability context compared with many other green events because they combine intensive personal investment (training and endurance), high public visibility, and strong collective rituals (shared routes, symbols, and community narratives). Such features can amplify emotional resonance and social identification, making sustainability-related self-definitions more salient than in low-involvement informational events. Therefore, green marathons provide a theoretically meaningful setting to examine how affective engagement precedes and enables identity-based motivation toward pro-sustainable behavior.

## 2 Literature review and research hypothesis

### 2.1 Green Marathon ecological awareness and pro-sustainable behavior tendency

In recent years, the Green Marathon, as a green event integrating the concept of environmental protection, has inspired participants’ ecological awareness and sense of environmental responsibility through the promotion of healthy lifestyles and the reinforcement of race scenarios (Tangporm et al., 2025). It has been pointed out that the relationship between ecological awareness and Pro-Sustainable Behavior Tendency is not linear, and that there is often an “awareness-behavior gap,” i.e., knowledge alone is not enough to translate into action in a stable manner, and that a combination of motivational and situational factors is needed to close the gap (Makkawan et al., 2025). From a more systematic theoretical perspective, Stern’s theory of environmentally salient behaviors and the “values-beliefs-norms” framework emphasize that environmental awareness can be further linked to behavioral dispositions through the internalization of values, beliefs and norms (Stern, 2000). Combined with empirical evidence from the Green Marathon context, the study found that marathon participants with higher eco-consciousness were more likely to show green donation intentions and a tendency to support sustainable measures for the event (Triantafyllidis and Kaplanidou, 2021). Accordingly, this study hypothesized that ecological awareness is positively related to Pro-Sustainable Behavior Tendency in the context of Green Marathon and proposed the following hypothesis:

*H1*: Green Marathon ecological awareness positively predicts pro-sustainable behavior tendency.

Environmental Value Integration can be understood as a process of individual understanding, perception and value internalization of environmental issues, which involves both cognitive assessment and may be accompanied by affective responses and attitude formation (Kanojia and Dhiman, 2025). In outdoor and nature settings, group participation and communal experiences tend to strengthen environmental attitudes and a sense of responsibility, thus providing contextual support for the transformation of ecological awareness into behavioral tendencies. Based on the “cognitive-emotional-intentional” pathway of the CAC theory, studies have found that environmental perceptions and emotional experiences (e.g., mobility experiences) are associated with more positive environmental attitudes and pro-sustainable behavioral intentions in tourists’ and nature recreation scenarios (Tang et al., 2022; Han, 2023). In natural contexts such as running, nature experiences are similarly associated with stronger environmental attitudes and Pro-Sustainable Behavior Tendency, and ecological awareness can be further correlated to the propensity to pay for sustainable measures such as carbon offsets (Triantafyllidis and Kaplanidou, 2018; Ólafsdóttir et al., 2021). In addition, the combination of nature connectedness and mobility experiences has been shown to be associated with greater environmental sensitivity and environmentally friendly behavior (Akçakese et al., 2024). Thus, ecological awareness integration may be positively associated with stronger emotional engagement and subsequent Pro-Sustainable Behavior Tendency during highly immersive nature events such as green marathons.

Green Behavior Belongingness refers to the social connection and group identity that individuals feel when participating in environmental protection actions, and this belonging experience is often associated with a stronger sense of environmental responsibility and Pro-Sustainable Behavior Tendency (O'Halloran, 2025). From a community perspective, shared goals and collective commitment can enhance individuals’ sense of identification with the group’s environmental actions and strengthen the sense of responsibility of “I am also an actor”. Empirical studies have shown that social satisfaction and a sense of belonging in outdoor activities can promote young people’s recognition of environmental responsibility and Pro-Sustainable Behavior Tendency (Zafeiroudi, 2020); Individuals’ sense of belonging and pro-environmental behavioral intentions are stronger when they feel that the group is working together for environmental goals (Reese and Junge, 2017); In addition, a collective public commitment program was also found to significantly enhance adolescent environmental behavior, suggesting that the mechanisms of belonging and commitment have a key role in promoting behavioral change (Lindemann-Matthies et al., 2021).

Collective Environmental Efficacy refers to the beliefs of group members that their collective actions are effective in improving environmental conditions, and such beliefs are usually associated with stronger environmental intentions and commitment to responsibility at the group level (Eunike et al., 2025). Under the CAC framework, collective efficacy can be viewed as a motivational cognition that is triggered by situational cues and interactional experiences, and may be further linked to behavioral intentions through emotional resonance and belongingness experiences. Previous studies have shown that Collective Environmental Efficacy is higher and more likely to be accompanied by environmental behaviors when individuals perceive that collective action can bring tangible environmental benefits (Chen et al., 2025). Collective identity and environmental identity have also been found to be closely related to environmental attitudes and related behavioral tendencies in urban contexts (Meis-Harris and Kashima, 2020). In addition, in the field of corporate environmental responsibility, collective-level factors such as shared vision enhance collective efficacy and are associated with green innovation and pro-sustainable behavior (Ruan et al., 2022). In events such as the Green Marathon, which emphasize team participation and public visibility, participants are more likely to develop the belief that “the collective can make an impact,” thus making the association between collective efficacy and Pro-Sustainable Behavior Tendency more prominent. Accordingly, the following hypotheses are proposed:

*H1a*: Environmental Value Integration Positively Influences Pro-Sustainable Behavior Tendency.

*H1b*: Green Behavior Belongingness Positively Influences Pro-Sustainable Behavior Tendency.

*H1c*: Collective Environmental Efficacy Positively Influences Pro-Sustainable Behavior Tendency.

The Green Marathon embeds the concept of sustainable development into sports participation and nature experience, so that participants are exposed to ecological cues and the atmosphere of collective action in a highly immersive context, thus providing a typical event context for emotional arousal and Environmental Empathy formation. It has been noted that environment-related issues have cross-level effects in social interaction contexts. For example, familiarity with climate issues among family members is not only related to individual pro-environmental behaviors, but also has a diffuse effect within the family through affective transfer and interaction mechanisms (Xia and Li, 2022). This finding suggests that environmental topics do not influence behavior only through cognitive pathways, but may also reinforce behavioral tendencies in social relationship networks through emotional resonance mechanisms. The role of emotional factors is more prominent in nature experience contexts. Research has shown that individuals who participate in nature-embedded sports such as trail running tend to form stronger nature connections and Environmental Empathy, and this emotional connection is further associated with pro-sustainable behavioral motivation (Grafnetterova, 2022). Similarly, recent empirical studies have pointed out that emotional reactions and feelings of responsibility in nature experiences significantly predict environmentally responsible behavioral intentions (Lu et al., 2025). Together, this evidence suggests that emotional experiences may serve as an important bridge between environmental perceptions and behavioral intentions in activities characterized by nature immersion and group participation. Therefore, in the Green Marathon, a context that integrates nature exposure, group interaction and sustainable communication, the Environmental Empathy generated by participants is more likely to strengthen their Pro-Sustainable Behavior Tendency. Based on this, the following hypotheses are proposed in this paper:

*H2*: Environmental empathy mediates the relationship between Green Marathon ecological awareness and pro-sustainable behavior tendency.

Individuals’ environmental cognition is not formed in isolation, but is continuously strengthened and emotionalized through social interactions and situational experiences. Research has shown that social incentives such as social relationships and trust can significantly enhance the effectiveness of sustainable behavioral interventions, not only in terms of information transfer, but also in terms of increasing individuals’ attention and emotional commitment to environmental issues (Nguyen-Van et al., 2021). That is, social interactions can transform cognitive-level environmental understanding into more motivationally colored behavioral dispositions through emotionally reinforcing pathways. In the family context, positive emotional climate and supportive interactions were significantly associated with stronger propensity for sustainable behavior, suggesting that emotional support can amplify the process of internalizing environmental values and make individuals more likely to experience Environmental Empathy (Corral-Verdugo et al., 2015). At the same time, observable “green behavioral cues” increase individuals’ attention to the consequences of their actions and influence subsequent behavioral choices by evoking moral emotions and a sense of responsibility (Topf and Speekenbrink, 2022). Together, this evidence suggests that social cues and situational cues do not act only at the cognitive level, but influence behavioral tendencies through affective mechanisms. In the Green Marathon, a highly interactive, publicly visible, and collective event that emphasizes sustainable communication, participants were both in the midst of social network interactions and exposed to a large number of observable green cues. This contextual structure may reinforce Environmental Empathy and make it easier for individuals to translate the experience of environmental responsibility into Pro-Sustainable Behavior Tendency.

In collective participation situations, individuals’ behavioral tendencies are not directly derived from cognitive judgments, but are reinforced through emotional mechanisms. It has been shown that the experience of identity and social connection brought about by group interaction can enhance individuals’ emotional engagement with environmental issues and increase their sense of responsibility and motivation for continued participation (Huang and Ali, 2025). When individuals experience recognition and acceptance in environmental actions, they are more likely to form emotional environmental resonance, thus increasing their subsequent Pro-Sustainable Behavior Tendency. Empirical evidence also suggests that pro-social tendencies are closely related to the experience of environmental identity and belonging, and that this identity internalization process is often accompanied by stronger environmental emotional commitment (Pérez Ibarra et al., 2020). This implies that the experience of belonging may further influence behavioral choices by enhancing Environmental Empathy. In contexts that emphasize common goals and collective tasks, group efficacy judgments may also play a role through affective pathways. Research has shown that collective efficacy is not only directly associated with pro-environmental intentions, but also with group identity and affective experiences (Plechatá et al., 2025; Valizadeh et al., 2025). The EIMECA model further suggests that environmental identity and affective factors have a key bridging role in collective action, connecting efficacy perceptions with behavioral tendencies (Carmona-Moya et al., 2021). Collective commitment and efficacy beliefs have also been found to reinforce environmental behavioral intentions through emotional resonance mechanisms in sport sustainability communication research (Trail and McCullough, 2020). In addition, positive thinking and attention to nature were shown to enhance nature connection and emotional empathy, thereby enhancing motivation for sustainable behavior, further supporting the mediating role of affective pathways in collective contexts (Poonamallee, 2025). Therefore, in activities with high interaction and strong common goals such as green marathons, group belonging experience and collective efficacy judgment are more likely to influence individuals’ Pro-Sustainable Behavior Tendency by reinforcing the affective mechanism of Environmental Empathy, which then influences individuals’ Pro-Sustainable Behavior Tendency. In other words, Environmental Empathy may constitute an important psychological bridge between identity experience and behavioral intention. Accordingly, the following assumptions are made:

*H2a*: Environmental Empathy Mediates Between Environmental Value Integration Influencing Pro-Sustainable Behavior Tendency.

*H2b*: Environmental Empathy Mediates the Role of Green Behavior Belongingness in Influencing Pro-Sustainable Behavior Tendency.

*H2c*: Environmental Empathy mediates the effect of Collective Environmental Efficacy on Pro-Sustainable Behavior Tendency.

In high-participation contexts such as green marathons, ecological awareness alone does not necessarily correspond to a long-term, stable propensity for sustainable behavior, and identity-related psychological mechanisms may play a key role (Tong et al., 2025). The study points out that the sustainable self-concept can serve as a source of intrinsic motivation for behavior, making individuals more likely to maintain consistent environmental behaviors across contexts (Haratbar et al., 2025). Meanwhile, identity cues and self-consistency mechanisms have also been shown to maintain or diffuse pro-environmental behaviors, e.g., by recalling and reminding past pro-environmental behaviors can strengthen identity and correlate with subsequent behavioral persistence (Fanghella et al., 2019). From an environmental education and normative perspective, the learning and reinforcement of ecologically responsible behaviors helps individuals to develop a more stable environmental identity, which in turn is associated with a propensity for sustainable behavior (Karvounidi et al., 2025). In addition, the internalization of personal values was found to be related to green purchasing intentions and motivations for environmental behaviors, providing a value-level explanation for the “awareness-identity-behavior” chain (Caniëls et al., 2021). Sports participation research also suggests that participation in activities with an eco-responsibility orientation is associated with stronger eco-self-identity and is further associated with a tendency to behave in an environmentally friendly manner (Tangporm et al., 2025). However, Self-Sustainable Behavior Identification (SSI) conceptually differs from these established constructs, such as environmental identity or green self-identity, by specifically emphasizing an individual’s identification with sustainable behaviors as part of their core self-concept, rather than a broader ecological or environmental orientation. Unlike environmental identity, which generally reflects a person’s general identification with environmental causes, SSI focuses more directly on how individual behaviors, particularly those linked to sustainability, are integrated into one’s self-definition and values system (Silintowe and Sukresna, 2022). SSI, therefore, acts as a more specific mechanism that bridges ecological awareness and behavior, making it distinct in its role as an intrinsic motivator for pro-sustainable behaviors (Gravelines et al., 2022). This distinction positions SSI uniquely within the identity theory literature, highlighting its role in behavior persistence and self-consistency, especially in the context of high-participation green events like the Green Marathon (Dermody et al., 2015). Accordingly, Self-Sustainable Behavior Identification (SSI) may serve as an important intentional dispositions mechanism connecting individuals’ ecological awareness and Pro-Sustainable Behavior Tendency in the context of Green Marathon and the following hypotheses are proposed:

*H3*: Self-sustainable identity mediates the relationship between Green Marathon ecological awareness and pro-sustainable behavior tendency.

In sustainable behavior research, there is growing evidence that whether individuals incorporate environmental roles into their self-concept is a key factor influencing behavioral stability and cross-situational consistency. Relevant studies have pointed out that green self-identity or green self-concept significantly predicts the propensity to consume sustainably and behave in an environmentally friendly manner, and that this effect is not simply an expression of attitude, but stems from self-consistency motives (Mishra et al., 2023). When individuals incorporate “I am an environmentalist” into their self-definition, their behaviors are more likely to be consistent across contexts. At the same time, environmental attitudes were found to bridge the gap between self-concept and responsible environmental behavior, suggesting that the “cognition-identity-behavioral disposition” pathway has important explanatory implications (Ojedokun and Balogun, 2010).

The process of identity internalization is particularly critical in collective contexts. Studies from the social identity and social exchange perspectives have shown that green identity at the group level predicts environmental citizenship behavior and influences individual spontaneous environmental actions through internalization mechanisms (Liu and Qi, 2022). Similarly, Green Self-Sustainability Identification plays a central role in Sustainable Consumption and Behavioral Intentions, while the experience of group belonging strengthens individuals’ identity congruence motives (Tung et al., 2017; Gravelines et al., 2022). Community identity research also points to the fact that shared goals and collective participation contexts contribute to increased internalization of identity and thus to increased environmental participation (Forsyth et al., 2015). In addition, the influence of climate beliefs and personal norms on environmental behavior was also found to work partly through the mechanism of environmental identity, further supporting the bridging role of identity in the “belief-behavioral disposition” pathway (Perera et al., 2022).

In situations involving group action, efficacy judgments may similarly influence behavior through identity mechanisms. Research has shown that individuals are more likely to be motivated to participate when they believe that group actions have real impacts, and that such beliefs can help to reinforce an individual’s self-positioning in relation to environmental roles (Ren et al., 2025). Efficacy beliefs may also serve as boundary conditions or reinforcers that translate established cognitive resources into actual behavioral tendencies (Vrselja et al., 2024). Social norms research has also shown that individuals are more likely to incorporate environmental roles into their self-concepts when situational cues reinforce the signal that “the group is acting” (Goldstein et al., 2008). In organizational contexts, collective responsibility climate was significantly associated with green identity and further predicted spontaneous environmental behavior, suggesting that collective-level efficacy and responsibility perceptions can influence behavioral choices through identity internalization mechanisms (Huang, 2024). Here, in the context of a Green Marathon, which is characterized by a high degree of public visibility, group interaction and common goals, Environmental Value Integration, Green Behavior Belonging Experience, and Collective Efficacy Judgment are more likely to enhance individuals’ Pro-Sustainable Behavior Tendency by reinforcing Self-Sustainable Behavior Identification (SSI), which enables them to internalize environmental roles into a stable self-concept.

*H3a*: Self-Sustainability Identification Mediates the Influence of Environmental Value Integration on Pro-Sustainable Behavior Tendency.

*H3b*: Self-Sustainable Belongingness Mediates the Influence of Green Behavior Belongingness on Pro-Sustainable Behavior Tendencies.

*H3c*: Self-Sustainability Identification Mediates the Influence of Collective Environmental Efficacy on Pro-Sustainable Behavior Tendency.

The CAC theory suggests that individual behavior is the result of the interaction of cognition (understanding and evaluation of external information), emotion (emotional response triggered by cognition) and volitional tendency (action tendency formed based on cognition and emotion) (Kanojia and Dhiman, 2025). The framework emphasizes that cognitive appraisals and affective experiences work together to shape intentional dispositions and are related to subsequent behavioral performance; at the same time, affective states may, in turn, influence cognitive judgments and thus the process of intention formation (Wang et al., 2025). Thus, CAC provides a concise and integrative theoretical perspective for explaining “how event-contextual cues translate into Pro-Sustainable Behavior Tendencies”.

**Figure 1:**
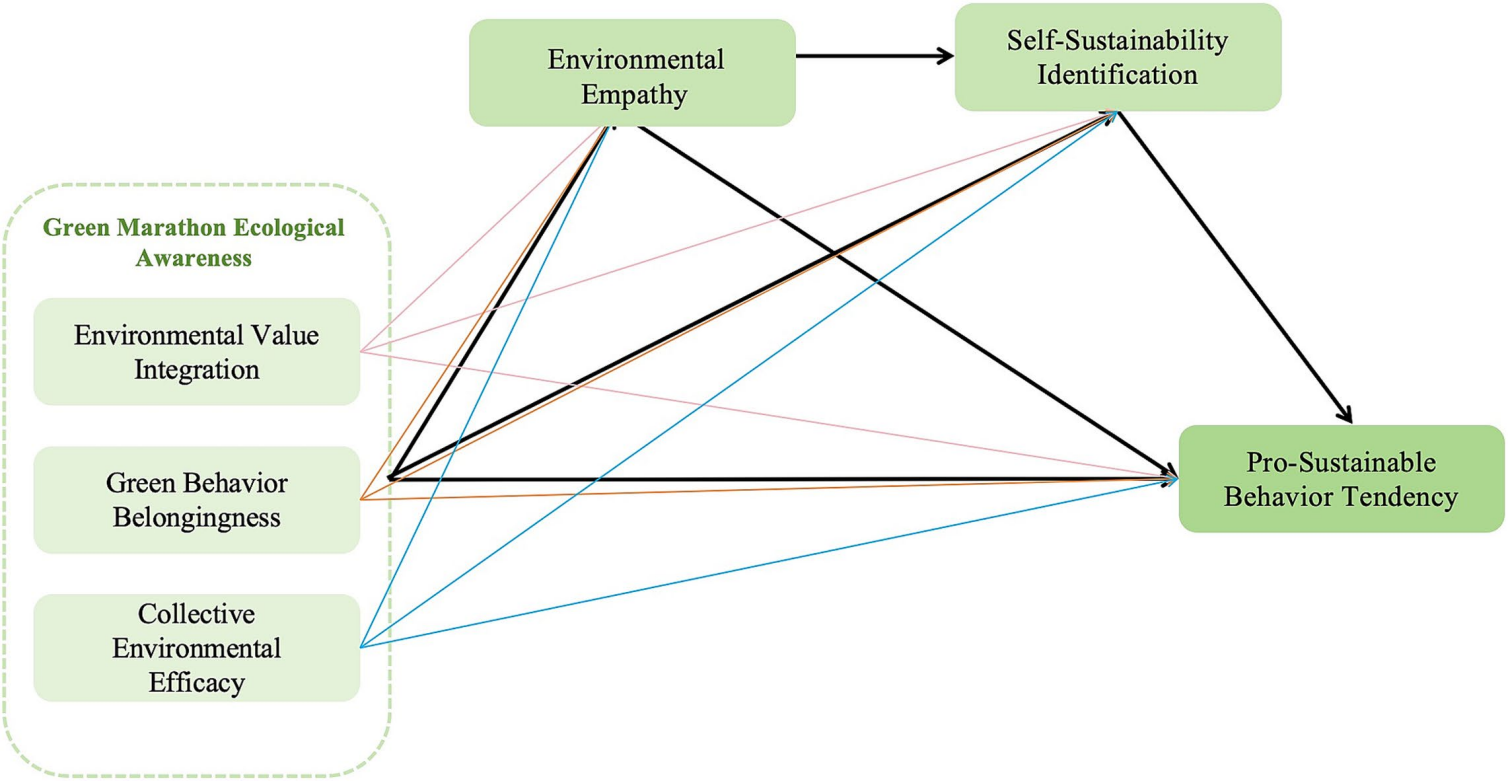
Proposed modeling diagrams.

Based on the CAC theory, this study corresponds Green Marathon Ecological Awareness to the cognitive dimension (participants’ understanding and evaluation of environmental issues), Environmental Empathy to the affective dimension (emotional empathy and care for the situation of nature and the environment), and Self-Sustainability Identification (SSI) to the intentional disposition dimension (the psychological tendency to incorporate “sustainability” into self-definition and to form action tendencies accordingly) (Wang et al., 2026) (the psychological tendency to incorporate “sustainability” into one’s self-definition and to form action tendencies accordingly). In the Green Marathon context, higher ecological awareness may be associated with stronger Environmental Empathy, which may further contribute to the internalization of sustainability into the self-definition (i.e., SSI) and make individuals more inclined to exhibit Pro-Sustainable Behavior Tendency. Based on this, this paper proposes a chain-mediated model (see Figure 1) that is consistent with CAC logic and the following hypotheses.

*H4*: Environmental empathy and self-sustainable identity sequentially mediate the relationship between Green Marathon ecological awareness and pro-sustainable behavior tendency.

*H4a*: Environmental empathy and self-sustainable identity sequentially mediate the relationship between environmental value integration and pro-sustainable behavior tendency.

*H4b*: Environmental Empathy and Self-Sustainability Belongingness mediate the chain between Green Behavior Belongingness and Pro-Sustainable Behavior Tendency.

*H4c*: Environmental Empathy and Self-Sustainability Belongingness mediate the chain between Green Behavior Belongingness and Pro-Sustainable Behavior Tendency.

## 3 Research design

### 3.1 Subjects and questionnaire distribution

This study employed a cross-sectional questionnaire design aimed at investigating the participation motivations and experiences of Green Marathon participants. The survey was conducted from January to May 2025. To ensure sample representativeness and data accuracy, the inclusion criteria for this study were strictly limited to individuals who had “participated in at least one Green Marathon event.” The research covered nine representative cities in China, including Kunming, Shanghai, Beijing, and Guangzhou, to capture the characteristics of participants from different regions. Questionnaire distribution was concentrated in high-traffic areas such as sports parks, public sports venues, and community activity centers in the aforementioned cities. Before inviting potential participants, researchers first screened them through key questions (e.g., “Have you ever participated in a Green Marathon event?”), and only distributed questionnaires to eligible Green Marathon participants.

The questionnaire was initially developed in English. To ensure the accuracy and cultural appropriateness of the Chinese version, this study employed standard translation and back-translation procedures. First, two bilingual public health researchers independently translated the English questionnaire into Chinese and negotiated to form a consolidated Chinese version. Subsequently, a translator with relevant research background who had not participated in the previous steps independently back-translated this Chinese version into English. Finally, the research team compared the back-translated version with the original English version item by item, discussing and revising entries with ambiguity or inconsistent expressions until the two versions achieved high consistency in semantics, concepts, and context, thereby ensuring the linguistic validity of the questionnaire.

Trained researchers conducted offline questionnaire distribution at selected locations in the aforementioned cities. After confirming that respondents met the inclusion criteria of “Green Marathon participants,” researchers clearly explained the research purpose to potential participants and emphasized the completely voluntary nature of participation. Questionnaires were distributed only after obtaining participants’ verbal consent. A total of 690 questionnaires were collected in this survey.

To ensure data quality, this study implemented strict data cleaning criteria. Exclusion criteria included: (1) questionnaires with more than 30% missing values; (2) questionnaires with highly consistent answers across all items; (3) questionnaires showing obvious patterned responses (such as “Z”-shaped or cyclic patterns). Through this process, 39 invalid questionnaires were removed, resulting in 651 valid samples, with an effective response rate of 94.3%.

This study strictly adhered to academic ethical standards throughout all stages of the research design. First, this study received approval from the Ethics Review Committee of Kunming Medical University (Approval Number: IPBKMU20250110). Before data collection began, all eligible research subjects were provided with written explanations containing detailed research content, data usage, confidentiality principles, and contact information. After ensuring each participant fully understood, we obtained their written informed consent. All participants were clearly informed that they had the right to withdraw from the study at any time unconditionally, without bearing any responsibility. To ensure data confidentiality, all collected questionnaires were anonymized, assigned only unique participant codes, and data were analyzed and reported only in aggregate form, stored in encrypted databases accessible only to core members of the research team.

### 3.2 Measurement of variables

A questionnaire survey was employed in this study. All measurement scales were adapted from established domestic and international scales and contextualized to the Green Marathon setting. A five-point Likert scale (1 = “Strongly Disagree” to 5 = “Strongly Agree”) was used for all items. The operational definitions, measurement items, and sources for each variable are presented in Table 1.

**Table 1:** Definition of variables and sources of reference scales.

Research variable	A test example of the question item	Reference scale
Environmental Value Integration (EVI)	I am more concerned about the environmental concepts of the Green Marathon event.	Perera et al. (2022) Ham et al. (2016)
Green Behavior Belongingness (GBB)	I feel part of a collective environmental campaign.	Hagerty and Patusky (1995) Malone et al. (2012)
Collective Environmental Efficacy (CEE)	Our team always manages to solve difficult environmental problems if we try hard enough.	Schwarzer et al. (1995) Cousineau et al. (2006)
Environmental Empathy (EE)	I am saddened and distressed by the damage done to the environment.	Davis (1983) Tam (2013)
Self-Sustainability Identification (SSI)	I am aware of my role and responsibility in promoting sustainability.	Phinney (1992) Kuswati et al. (2021)
Pro-Sustainable Behavior Tendency (PSBT)	I will continue to adopt a sustainable lifestyle after the Green Marathon.	Tapia-Fonllem et al. (2013) Hines et al. (1987)

Environmental Value Integration (EVI) measures the degree to which individuals identify with and internalize the environmental concepts conveyed by the Green Marathon event. This variable emphasizes whether participants perceive the event as a vehicle for transmitting environmental values, rather than merely a sporting competition. Six items were adapted from Ballantyne et al. (2008) and Ham et al. (2016). A sample item is: “I am more concerned about the environmental concepts of the Green Marathon event.”

Green Behavior Belongingness (GBB) measures the psychological connection and sense of belonging individuals perceive when participating in collective pro-environmental actions. Grounded in social identity theory, this construct captures the sense of “we-ness” formed through shared activities. Eight items were adapted from Hagerty and Patusky (1995) and Malone et al. (2012). A sample item is: “I feel part of a collective environmental campaign.”

Collective Environmental Efficacy (CEE) measures an individual’s belief in the capability of their team to collectively solve environmental problems. Distinguished from self-efficacy, collective efficacy focuses on group-level confidence (“we can do it”). Ten items were adapted from Schwarzer et al. (1995) and Cousineau et al. (2006). A sample item is: “Our team always manages to solve difficult environmental problems if we try hard enough.”

Environmental Empathy (EE) measures an individual’s affective responses—including sympathy, distress, and concern—to damage inflicted upon the natural environment. Eight items were adapted from Davis (1983) and Tam (2013). A sample item is: “I am saddened and distressed by the damage done to the environment.”

Self-Sustainable Identity (SSI) measures an individual’s role awareness and sense of responsibility in promoting sustainable development. This construct is distinct from a generalized environmental identity (which emphasizes “my connection with nature”). Instead, SSI focuses on the degree to which individuals internalize the social role of “I am a promoter of sustainability,” emphasizing the transition from environmental concern to the assumption of responsibility. Five items were contextually developed based on Phinney (1992) identity development theory and Kuswati et al. (2021) research on sustainable consumption identity. A sample item is: “I am aware of my role and responsibility in promoting sustainability.”

Pro-Sustainable Behavior Tendency (PSBT) measures an individual’s intention to continue adopting a sustainable lifestyle after participating in the Green Marathon. According to attitude-behavior theory, behavioral tendency is the strongest proximal antecedent of actual behavior. Five items were adapted from Tapia-Fonllem et al. (2013) and Hines et al. (1987). A sample item is: “I will continue to adopt a sustainable lifestyle after the Green Marathon.”

To facilitate repeated measurements, this paper provides the complete measurement questionnaire, as shown in Appendix A.

## 4 Data analysis

### 4.1 Descriptive statistics

The study utilized a questionnaire to collect data from participants of different backgrounds, including age, gender, education level, income level, and level of concern for environmental issues. By analyzing the frequency distribution of these data, we were able to depict the basic characteristics of the participants and further explore how these characteristics correlate with the number of times they participated in the Green Marathon.

The age distribution showed that the largest number of participants were aged 26–35 years, accounting for 57.6% of the total sample. The gender ratio was close, with 54% male and 46% female. Education level was dominated by bachelor’s degree, accounting for 50.9%, followed by college and high school and below. The income level was mainly concentrated in the range of 5,000–10,000 yuan, accounting for 45.2%.

Degree of concern for environmental issues: The largest number of participants, 258, or 40.1%, were very concerned about environmental issues, indicating that a significant portion of the population is highly concerned about environmental issues. The number of participants who were concerned about environmental issues was 234, accounting for 36.3%. The number of participants who are generally concerned about environmental issues is 97, accounting for 15.1%. The least number of participants who are not concerned about environmental issues is 55, accounting for 8.5%, which may be related to the fact that this part of the population does not have enough knowledge about or is not interested in environmental issues.

Number of times participating in the Green Marathon: The largest number of participants have participated in the Green Marathon 1–2 times, with 424 participants, accounting for 65.8%, which may indicate that the Green Marathon activity has a greater attraction to first-time participants. The number of participants who have participated in 3–5 times is 182, accounting for 28.3%, which shows that some of the participants have some loyalty to the Green Marathon activities. The detailed table is shown in Table 2.

**Table 2:** General frequency distribution table (*N* = 644).

Theme	Options	Frequency (n)	Percentage (%)
Age	18–25 years	1	0.2
	26–35 years	371	57.6
	36–45 years	190	29.5
	46 and over	82	12.7
Gender	Male	348	54.0
	Female	296	46.0
Educational level	High school and below	42	6.50
	Associate degree	146	22.7
	Bachelor’s degree	328	50.9
	Master’s degree	99	15.4
	PhD and above	29	4.50
Monthly salary	3,000–5,000 yuan	94	14.6
	5,000–8,000 yuan	291	45.2
	8,000–15,000 yuan	175	27.2
	15,000 and above	84	13.0
Self-assessment of environmental concern	Not interested	55	8.5
	General	97	15.1
	Concern	234	36.3
	Deep concern	258	40.1
Participation in the Green Marathon	1–2 times	424	65.8
	3–5 times	182	28.3
	6 or more	38	5.9

Income is reported in Chinese yuan (CNY).

### 4.2 Reliability

In this paper, the Cronbach ‘s alpha reliability coefficient method is used to test the reliability of the sample, resulting in a Cronbach ‘s alpha coefficient of 0.946 which is greater than 0.7, which synthesizes to show that the data reliability is of high quality and can be used for the next step of analysis. The Cronbach ‘s alpha coefficient for the giant dimension is shown in Table 3.

**Table 3:** Confidence analysis table.

Dimension	Number of measurement items	Cronbach alpha
Overall questionnaire	42	0.946
EVI	6	0.910
GBB	8	0.905
CEE	10	0.932
EE	8	0.908
SSI	5	0.873
PSBT	5	0.844

### 4.3 Validity

The validity of the questionnaire in this study was mainly tested by KMO (Kaiser-Meyer-Olkin) test and validated factor analysis. In this study, the value of KMO is 0.951, which indicates excellent validity, and the results are shown in Table 4.

**Table 4:** KMO test and Bartlett’s test of sphericity results.

Dimension	KMO value	Bartlett’s test of sphericity	
		chi-square value	*P*
Overall questionnaire	0.951	15681.481	0.000
EVI	0.921	2277.751	0.000
GBB	0.932	2590.355	0.000
CEE	0.963	3861.898	0.000
EE	0.947	2623.029	0.000
SSI	0.880	1466.155	0.000
PSBT	0.866	1172.545	0.000

### 4.4 Exploratory factor analysis

To examine the underlying structure of the measurement scale, an exploratory factor analysis (EFA) was conducted on the 42 items using principal component analysis with Varimax rotation. The Kaiser–Meyer–Olkin (KMO) measure of sampling adequacy was 0.951, exceeding the recommended threshold of 0.70, and Bartlett’s test of sphericity was significant (*χ*^2^ = 15681.481, *p* < 0.001), indicating that the data were suitable for factor analysis.

Based on the criterion of eigenvalues greater than 1, six factors were extracted, collectively accounting for 63.36% of the total variance (see Table 5). The rotated factor matrix (see Table 6) demonstrated that all items loaded cleanly onto their respective factors, with factor loadings exceeding 0.60 and no significant cross-loadings. No items were deleted during this process, as all 42 original items demonstrated satisfactory psychometric properties. The clean factor structure and high loadings confirm that the 42-item scale demonstrates good construct validity. The cumulative variance explained (63.36%) exceeds the recommended threshold of 60%, indicating that the six factors adequately capture the underlying constructs. The detailed results of the total variance explained and the rotated component matrix are presented in Tables 5, 6, respectively.

**Table 5:** Total variance explained.

Ingredient	Initial eigenvalue			Extract the sum of the squares of the loads			Rotational load sum of squares		
	Grand total	Percentage of variance	Cumulative %	Grand total	Percentage of variance	Cumulative %	Grand total	Percentage of variance	Cumulative %
1	13.211	31.456	31.456	13.211	31.456	31.456	6.320	15.047	15.047
2	3.723	8.864	40.320	3.723	8.864	40.320	4.943	11.768	26.816
3	3.021	7.193	47.513	3.021	7.193	47.513	4.682	11.148	37.964
4	2.656	6.323	53.837	2.656	6.323	53.837	4.167	9.923	47.887
5	2.109	5.021	58.858	2.109	5.021	58.858	3.359	7.998	55.884
6	1.891	4.502	63.360	1.891	4.502	63.360	3.140	7.476	63.360

**Table 6:** Analyzed table after rotation.

	Ingredient					
	1	2	3	4	5	6
CEE1	0.776					
CEE2	0.765					
CEE5	0.765					
CEE6	0.754					
CEE8	0.752					
CEE9	0.743					
CEE3	0.743					
CEE7	0.742					
CEE10	0.741					
CEE4	0.732					
GBB2		0.777				
GBB5		0.741				
GBB7		0.738				
GBB3		0.737				
GBB8		0.733				
GBB6		0.733				
GBB1		0.732				
GBB4		0.722				
EE2			0.707			
EE3			0.700			
EE1			0.699			
EE6			0.698			
EE8			0.697			
EE7			0.692			
EE4			0.690			
EE5			0.675			
EVI1				0.812		
EVI5				0.790		
EVI4				0.772		
EVI6				0.771		
EVI3				0.764		
EVI2				0.762		
SSI1					0.784	
SSI2					0.768	
SSI4					0.762	
SSI3					0.758	
SSI5					0.744	
PSBT2						0.766
PSBT4						0.752
PSBT5						0.742
PSBT1						0.718
PSBT3						0.716

Extraction method: principal component analysis.

Rotation method: Kaiser normalized maximum variance method.

### 4.5 Common methodological deviations

Given that all data were collected through self-reported questionnaires from a single source at one point in time, there is a potential risk of common method bias (CMB) that may artificially inflate relationships among variables (Podsakoff et al., 2003). To assess the severity of CMB, we employed the confirmatory factor analysis (CFA) marker technique, comparing the fit of a single-factor model (where all 42 items were loaded onto one latent factor) with that of the hypothesized six-factor measurement model.

As shown in Table 7, the single-factor model exhibited poor fit: *χ*^2^(303) = 8232.063, *χ*^2^/df = 10.051, RMSEA = 0.119, GFI = 0.457, AGFI = 0.401, CFI = 0.512, NFI = 0.487. In contrast, the six-factor measurement model demonstrated excellent fit: *χ*^2^(303) = 1215.427, *χ*^2^/df = 1.512, RMSEA = 0.028, GFI = 0.918, AGFI = 0.908, CFI = 0.973, NFI = 0.924. The substantial improvement in fit indices from the single-factor to the multi-factor model suggests that common method bias is not a serious concern in this study.

**Table 7:** Comparison of fit indices.

Fit index	Single-factor model	Measurement model
*χ*^2^	8232.063	1215.427
*χ*^2^/df	10.051	1.512
RMSEA	0.119	0.028
GFI	0.457	0.918
NFI	0.487	0.924
AGFI	0.401	0.908
CFI	0.512	0.973

Recommended cut-off values for a good model fit are: *χ*^2^/df < 3, RMSEA < 0.05, GFI > 0.90, NFI > 0.90, AGFI > 0.90, CFI > 0.90.

### 4.6 Validation factor analysis

It should be noted that both the exploratory factor analysis (EFA) and confirmatory factor analysis (CFA) were conducted on the same sample. This approach was adopted for two reasons. First, given the contextual adaptation of certain scales (particularly the Self-Sustainable Identity construct) to the Green Marathon setting, EFA was necessary to empirically verify the factor structure before proceeding to CFA. Second, the large sample size (*N* = 644) exceeds the recommended threshold for conducting both analyses on the same sample, and the stable factor structure observed in EFA (with all factor loadings > 0.60 and no cross-loadings) provided confidence for subsequent CFA. However, we acknowledge that conducting both analyses on the same sample may lead to capitalizing on chance characteristics and potentially inflate model fit indices. To mitigate this concern, we ensured that the CFA model was specified *a priori* based on theoretical foundations rather than solely on EFA results, and we employed multiple fit indices to rigorously evaluate model fit. The excellent fit of the measurement model suggests that the factor structure is robust and not merely an artifact of sample-specific variation.

In order to further verify whether the questionnaire is consistent with the expected theory, and to examine whether a measurement item has a significant loading with the corresponding factor but not with the irrelevant factor, AMOS26.0 software was used to conduct a validation factor analysis of the questionnaire. First, based on the previous relevant theory, the validation factor analysis model was constructed and imported into the previous SPSS sample data, followed by judging the model fit. As can be seen from the table of model fitting coefficients, the chi-square degrees of freedom ratio *χ*^2^/df value of 1.512 is less than 5, and the fit is ideal; the RMSEA is 0.028 less than 0.05, and the fit is ideal; the GFI is 0.918, the NFI is 0.924, the CFI is 0.973, the AGFI is 0.908, and all the above indexes reach the standard of 0.9, and the result is a good fit. Therefore, the comprehensive judgment of the model fit is good. The table of model fitting coefficients is shown in Table 8.

**Table 8:** Table of model fitting coefficients.

Commonly used indicators	*χ*^2^/df	RMSEA	GFI	CFI	NFI	AGFI
Standard of judgment	<3	<0.05	>0.9	>0.9	>0.9	>0.9
Value	1.512	0.028	0.918	0.973	0.924	0.908

Convergent validity refers to whether the observed variables measuring the same latent variable converge to that latent variable. In this study, the aggregation validity of the model was tested using Factor Loading, Average Variance Extracted (AVE), and Composite Reliability (CR). It is generally believed that the aggregation validity of the questionnaire is better when the AVE is greater than 0.5 and the CR is greater than 0.6 or more.

According to the model validity table, the (standardized) factor loadings of the 42 measurement items in this study were all above 0.6, with the vast majority above 0.7; the AVE for each variable were all greater than 0.5 and the CR were all greater than in the range of 0.845–0.932, with the vast majority above 0.7 (see Tables 9, 10). This indicates that the study has high convergent validity.

**Table 9:** Model validity table.

Path			Estimate
GBB8	←	GBB	0.725
GBB7	←	GBB	0.735
GBB6	←	GBB	0.713
GBB5	←	GBB	0.719
GBB4	←	GBB	0.750
GBB3	←	GBB	0.753
GBB2	←	GBB	0.772
GBB1	←	GBB	0.732
EVI6	←	EVI	0.771
EVI5	←	EVI	0.810
EVI4	←	EVI	0.777
EVI3	←	EVI	0.799
EVI2	←	EVI	0.776
EVI1	←	EVI	0.823
CEE10	←	CEE	0.766
CEE9	←	CEE	0.753
CEE8	←	CEE	0.758
CEE7	←	CEE	0.760
CEE6	←	CEE	0.753
CEE5	←	CEE	0.779
CEE4	←	CEE	0.770
CEE3	←	CEE	0.728
CEE2	←	CEE	0.755
CEE1	←	CEE	0.786
EE1	←	EE	0.787
EE2	←	EE	0.754
EE3	←	EE	0.770
EE4	←	EE	0.716
EE5	←	EE	0.729
EE6	←	EE	0.740
EE7	←	EE	0.716
EE8	←	EE	0.739
SSI1	←	SSI	0.796
SSI2	←	SSI	0.784
SSI3	←	SSI	0.748
SSI4	←	SSI	0.746
SSI5	←	SSI	0.736
PSBT1	←	PSBT	0.748
PSBT2	←	PSBT	0.735
PSBT3	←	PSBT	0.688
PSBT4	←	PSBT	0.722
PSBT5	←	PSBT	0.718

**Table 10:** Composite reliability and convergent validity.

Variable	CR	AVE
GBB	0.905	0.544
EVI	0.910	0.629
CEE	0.932	0.579
EE	0.909	0.554
SSI	0.874	0.581
PSBT	0.845	0.522
GBB	0.905	0.544

CR, composite reliability; AVE, average variance extracted. Acceptable thresholds are CR > 0.70 and AVE > 0.50. All constructs in this study demonstrate adequate composite reliability and convergent validity.

That is, the scale has differential validity when the AVE square root value of each variable is greater than its correlation coefficient with each of the other variables. As shown in Table 11, the AVE square root value of each variable (main diagonal part) is greater than the correlation coefficient between the variable and other variables, indicating that all dimensions of the formal scale questionnaire have reached the criterion of discriminant validity and passed the test of discriminant validity, and the formal scale questionnaire can be constructed for structural equation modeling.

**Table 11:** Distinguishing validity scale.

	AVE	PSBT	EVI	GBB	CEE	EE	SSI
PSBT	0.522	0.722					
EVI	0.629	0.329	0.793				
GBB	0.544	0.311	0.359	0.738			
CEE	0.579	0.322	0.361	0.338	0.761		
EE	0.554	0.457	0.479	0.442	0.489	0.744	
SSI	0.581	0.345	0.323	0.323	0.324	0.483	0.762

Diagonal values represent the square root of AVE.

### 4.7 Correlation analysis

In this study, we conducted a correlation analysis between the pro-environmental behavioral tendencies of Green Marathon participants and related psychological variables to investigate how green marathons promote pro-environmental behavior through environmental awareness, environmental empathy, and self-sustainable identity. The following is a detailed description of the results of the correlation analysis between the independent variables (environmental value integration, green behavior belongingness, and collective environmental efficacy) and the dependent variable (pro-sustainable behavior tendency). The results of the correlation analysis are shown in Table 12.

**Table 12:** Correlation analysis results table.

	PSBT	EVI	GBB	CEE	EE	SSI
PSBT	—					
EVI	0.329**	—				
GBB	0.311**	0.359**	—			
CEE	0.322**	0.361**	0.338**	—		
EE	0.457**	0.479**	0.442**	0.489**	—	
SSI	0.345**	0.323**	0.323**	0.324**	0.483**	—

**p < 0.01.

### 4.8 Structural equation modeling

**Figure 2:**
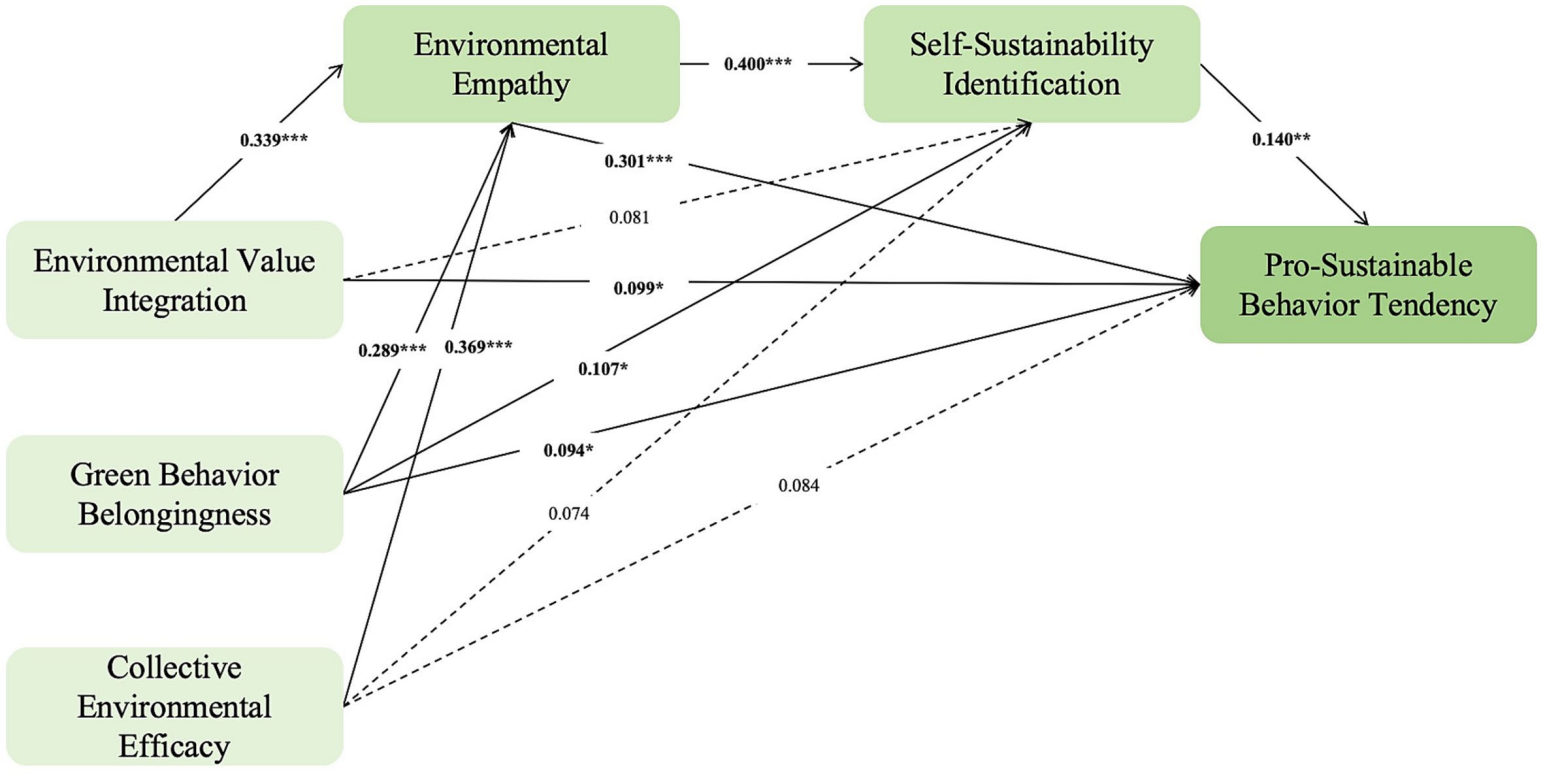
Plot of the results of the modified structural equations. *p < 0.05, **p < 0.01, ***p < 0.001.

This study utilized AMOS 26.0 to construct a research model to test the fit of this research model and also to validate the research hypotheses in this study. It aims to investigate how Green Marathon affects pro-sustainable behavior tendency through three independent variables, namely, environmental value integration, green behavior belongingness, and collective environmental efficacy, and two mediating variables, namely, environmental empathy and self-sustainable identification. In this section, the hypothesized paths are analyzed for significance based on the results of the structural equation modeling runs, and the results of the analysis are as follows: A summary of the model parameter estimates is shown in Table 12. The final modified structural equation modeling diagram of this paper is shown in Figure 2.

### 4.9 Analysis of mediating effects

To test the significance of the mediation effects, a bias-corrected bootstrapping procedure with 5,000 resamples was employed following Preacher and Hayes (Preacher and Hayes, 2008) (Table 13).

**Table 13:** Summary table of model parameter estimates.

			Unstandardized estimate	S. E.	C. R.	*P*	Standardized estimate (β)
EE	←	EVI	0.265	0.032	8.374	***	0.339
EE	←	GBB	0.260	0.036	7.203	***	0.289
EE	←	CEE	0.314	0.035	9.073	***	0.369
SSI	←	EVI	0.074	0.040	1.841	0.066	0.081
SSI	←	GBB	0.112	0.045	2.479	*	0.107
SSI	←	CEE	0.073	0.044	1.677	0.094	0.074
SSI	←	EE	0.466	0.064	7.315	***	0.400
PSBT	←	EVI	0.081	0.037	2.195	*	0.099
PSBT	←	GBB	0.088	0.042	2.109	*	0.094
PSBT	←	CEE	0.074	0.040	1.853	0.064	0.084
PSBT	←	EE	0.315	0.062	5.097	***	0.301
PSBT	←	SSI	0.126	0.045	2.790	**	0.140

***p < 0.001, **p < 0.01, *p < 0.05. Non-significant paths (*p* > 0.05) are reported with their exact *p*-values.

Unstandardized effect estimates (B) are reported along with their 95% confidence intervals; effects are considered significant if the confidence interval does not include zero (see Table 14).

**Table 14:** Mediated effects analysis table.

	Effect estimate (B)	SE	*Z*	Lower (95% CI)	Upper (95% CI)
CEE-PSBT total effect	0.201	0.045	4.467	0.118	0.295
CEE-PSBT direct effect	0.074	0.047	1.574	−0.012	0.171
CEE-PSBT total indirect effects	0.126	0.027	4.667	0.079	0.186
CEE-EE-SSI-PSBT indirect effect	0.018	0.008	2.250	0.005	0.039
CEE-EE-PSBT indirect effect	0.099	0.025	3.960	0.055	0.155
CEE-SSI-PSBT indirect effect	0.009	0.007	1.286	−0.001	0.030
EVI-PSBT total effect	0.190	0.043	4.419	0.103	0.275
EVI-PSBT direct effect	0.074	0.047	1.574	−0.010	0.171
EVI-PSBT total indirect effect	0.108	0.022	4.909	0.070	0.156
EVI-EE-SSI-PSBT indirect effect	0.016	0.007	2.286	0.004	0.032
EVI-EE-PSBT indirect effect	0.083	0.021	3.952	0.047	0.130
EVI-SSI-PSBT indirect effect	0.009	0.008	1.125	−0.002	0.031
GBB-PSBT total effect	0.199	0.050	3.980	0.105	0.303
GBB-PSBT direct effect	0.088	0.047	1.872	0.000	0.183
GBB-PSBT total indirect effects	0.111	0.025	4.440	0.070	0.168
GBB-EE-SSI-PSBT indirect effect	0.015	0.007	2.143	0.004	0.032
GBB-EE-PSBT indirect effect	0.082	0.023	3.565	0.044	0.133
GBB-SSI-PSBT indirect effect	0.014	0.010	1.400	0.001	0.043

Confidence intervals are bias-corrected bootstrap (5,000 samples).

#### 4.9.1 Collective environmental efficacy (CEE)

The total effect of CEE on pro-sustainable behavior tendency was significant (*B* = 0.201, 95% CI: [0.118, 0.295]). The direct effect was not significant (*B* = 0.074, 95% CI: [−0.012, 0.171]), while the total indirect effect was significant (*B* = 0.126, 95% CI: [0.079, 0.186]). Examining the specific indirect paths, the chain mediation path through environmental empathy and self-sustainable identity (CEE → EE → SSI → PSBT) was significant (*B* = 0.018, 95% CI: [0.005, 0.039]), as was the single mediation path through environmental empathy alone (CEE → EE → PSBT: *B* = 0.099, 95% CI: [0.055, 0.155]). However, the path through self-sustainable identity alone (CEE → SSI → PSBT) was not significant (*B* = 0.009, 95% CI: [−0.001, 0.030]). In practical terms, this suggests that participants’ belief in their group’s environmental capability primarily translates into behavioral intentions by first evoking an emotional response; without this emotional engagement, collective efficacy alone does not foster a lasting sustainable identity or directly influence behavior.

#### 4.9.2 Environmental value integration (EVI)

The total effect of EVI on pro-sustainable behavior tendency was significant (*B* = 0.190, 95% CI: [0.103, 0.275]). The direct effect was not significant (*B* = 0.074, 95% CI: [−0.010, 0.171]), while the total indirect effect was significant (*B* = 0.108, 95% CI: [0.070, 0.156]). The chain mediation effect (EVI → EE → SSI → PSBT: *B* = 0.016, 95% CI: [0.004, 0.032]) and the mediation effect through EE alone (EVI → EE → PSBT: *B* = 0.083, 95% CI: [0.047, 0.130]) were both significant, whereas the mediation effect through SSI alone (EVI → SSI → PSBT: *B* = 0.009, 95% CI: [−0.002, 0.031]) was not. This indicates that cognitive awareness of environmental values must be emotionally charged to become personally relevant; intellectual understanding alone is insufficient to shape one’s sustainable identity or drive behavior.

#### 4.9.3 Green behavior belongingness (GBB)

The total effect of GBB on pro-sustainable behavior tendency was significant (*B* = 0.199, 95% CI: [0.105, 0.303]). The direct effect was also significant (*B* = 0.088, 95% CI: [0.000, 0.183]), as was the total indirect effect (*B* = 0.111, 95% CI: [0.070, 0.168]). Furthermore, the chain mediation effect (GBB → EE → SSI → PSBT: *B* = 0.015, 95% CI: [0.004, 0.032]), the mediation effect through EE alone (GBB → EE → PSBT: *B* = 0.082, 95% CI: [0.044, 0.133]), and the mediation effect through SSI alone (GBB → SSI → PSBT: *B* = 0.014, 95% CI: [0.001, 0.043]) were all significant. Unlike the other two awareness dimensions, a sense of belonging to a green community has both direct and indirect effects on behavior. In practice, this means that fostering a collective identity among participants is particularly powerful—it can influence behavioral intentions both through emotional pathways and by directly reinforcing one’s self-concept as a sustainability practitioner.

## 5 Discussion

### 5.1 Two separate mediating paths of self-sustainable identity are not significant

Our findings contribute to the literature in three ways. First, the results support a sequential mechanism from awareness to empathy to self-sustainable identity to pro-sustainable behavior, suggesting that emotional engagement is a key bridge between cognitive recognition and downstream volitional tendencies. This extends prior studies that often emphasize either empathy-driven behavior or identity-driven behavior by empirically testing an ordered process linking both. Second, the Green Marathon context appears to provide a psychologically distinctive setting in which sustainability-related cues and collective participation can make self-definitions more salient, thereby facilitating identity-based motivation. Third, viewed through CAC theory, the evidence implies that the conative component may be more meaningfully captured by identity-based volition in event contexts, and that cognition may not translate into conation without affective activation—highlighting a pathway-level refinement rather than treating CAC as a purely linear assumption.

One possible explanation for the non-significance of self-sustainable identity in the pathway of environmental value integration influencing the propensity to behave sustainably is the absence of cognitive nature of environmental awareness integration with affective mechanisms. In the Green Marathon setting, environmental value integration may include knowledge acquisition about issues such as climate change, plastic pollution, and ecological conservation, as well as attitudes and beliefs about these issues. This is a rational, cognitive-level process, and while it helps to increase an individual’s environmental awareness, it lacks a strong affective motivation on its own. Self-sustainable identity, on the other hand, is a deeper psychological construction that goes beyond cognitive acceptance and is the result of an individual’s close connection between environmental behavior and his or her own identity, values, and lifestyle. In other words, self-sustainable identity is more of an emotional and behavioral identification, a transformation process of “environmental behavior is part of me”. However, in the context of a Green Marathon, environmental value integration as a cognitive response may not directly stimulate self-sustainable identity. Although participants may be aware of the need to protect the environment, if these perceptions are not linked to a stronger identity through emotional engagement, it is difficult to directly transform them into environmental behaviors. Therefore, environmental value integration may remain at the cognitive level only, and may not be deeply internalized into sustainable behaviors through self-sustainable identity (Arman and Mark-Herbert, 2022).

The path in which collective environmental efficacy is associated with the propensity for sustainable behavior is similarly insignificant. One possible explanation for this is the lack of connection between collective environmental efficacy and affective identity. In the pathway of this study, collective environmental efficacy failed to significantly be associated with pro-sustainable behavior tendency through self-sustainable identification, most notably because collective efficacy, as a cognitive belief, does not sufficiently stimulate an individual’s affective response. Even if collective action is effective, an individual’s perception of collective efficacy does not necessarily translate directly into personal-level affective identification. Thus, collective efficacy did not directly be associated with sustainable behavior through self-sustainable identity. In contrast, environmental empathy, as an affective driver, can break this cognitive limitation. Environmental empathy triggers individuals’ emotional response, which makes collective environmental efficacy not only stay at the cognitive level, but also transforms into emotional identification of environmental behaviors, which is associated with individuals’ self-sustainable identity and ultimately is associated with their behaviors.

Taken together, these non-significant direct paths point to a broader theoretical insight: within the CAC framework, cognitive appraisals alone, whether about personal values or collective capabilities, appear insufficient to shape identity or behavioral intentions without affective processing. In the Green Marathon context, participants may cognitively recognize environmental values and believe in their group’s collective power, yet these cognitive resources seem to remain psychologically inert unless activated by emotional engagement. This may help explain why environmental empathy occupies such a central position in our model, serving as the critical gateway through which abstract awareness becomes personally meaningful, transforming “knowing” into “being” and, ultimately, into “doing.” For event organizers and sustainability practitioners, this suggests that interventions focused solely on raising awareness or building collective confidence may fall short; cultivating emotional connection to environmental issues is not optional but essential for translating cognitive beliefs into lasting behavioral commitments.

### 5.2 Chain intermediation

In the Green Marathon context, environmental value integration can be effectively translated into behavioral motivation through emotional responses. Individuals’ awareness of environmental issues (e.g., understanding the dangers of plastic pollution or carbon emissions) does not stop at the rational level, but triggers emotional empathy during the event, especially when they see the environmental benefits of the collective activity. Environmental empathy thus becomes an important emotional mediator, pushing individuals from rational understanding to emotional identification. This emotional identification further motivates individuals to closely integrate environmental behaviors with their self-image to form a self-sustainable identity, making environmental actions part of their identity. Ultimately, this identity is linked to individuals to show stronger pro-sustainable behavior tendency in their future behaviors. At the same time, when participants felt that they were not just running for personal achievement, but were contributing to a larger environmental goal, this sense of belonging stimulated their environmental empathy. They began to feel emotionally responsible for environmental issues, which strengthened their identification with environmental action. This identification in turn further shapes the individual’s self-sustainable identity, making environmental protection part of the individual’s self-worth. This is because when individuals view environmental actions as part of their identity, they are more inclined to consistently demonstrate pro-environmental behaviors in their future actions.

Collective environmental efforts (e.g., working together to reduce the use of plastic bottles, promoting green running, etc.) be associated with participants’ emotional resonance through collective efficacy. Participants’ environmental empathy is triggered when they see the collective’s environmental successes, thus identifying with their own responsibility as part of the environmental action. Emotional empathy helps individuals be associated with their environmental self-identity at the cognitive level, i.e., they are not only participants but also practitioners of environmental behaviors. Ultimately, this identification is linked to individuals to adopt more positive environmental behaviors. Similarly, Schneider and van der Linden’s research suggests that “sustainable emotions” can create positive feedback loops that encourage individuals to adopt more environmentally friendly practices. Emotions such as awe, pride, and responsibility can inspire actions that directly support sustainability efforts, while also helping to increase an individual’s environmental awareness (Schneider and van der Linden, 2023).

### 5.3 Comparison of studies

Like Schultz and Zelezny (1998) study, many studies do suggest that emotional empathy is critical to the be associated with environmental behavior. However not all literature on the interlocking roles of environmental empathy and self-sustainable identity as mediators has been focused on. In some studies, environmental empathy and identification have been analyzed as independent mediating variables rather than connecting cognition, emotion, and behavior in a chained fashion, e.g., Gifford (2007) and Carfora et al. (2017). whereas this paper marks a more novel perspective by suggesting that the relationship between the two factors is mediated through a chain. Not coincidentally, this paper proposes the role of environmental empathy and self-sustainable identity as chain mediators between green behavior belongingness influencing pro-sustainable behavior tendency. Belongingness has indeed been mentioned in some green behavior studies, e.g., Steg and Vlek (2009) emphasize that an increased sense of belongingness of an individual is associated with the persistence of environmental behaviors. However, the interlocking effects of green behavior belongingness in combination with affective identification, especially in combination with the path of self-sustainable identity, may not have been explored in detail in a large number of studies. Most studies may have focused more on the relationship between the sense of efficacy of group actions and the sense of belonging to collective behaviors, while fewer comprehensive analyses have been conducted at the level of individual affective identity. This paper proposes a multilevel, multifactorial analytic framework, which is not common in the existing literature. Most existing studies are more likely to explore the direct relationship between environmental empathy and behavior, or the single role of emotional identity, this paper innovates based on the results of previous studies and builds this model based on the CAC theory. Meanwhile, the investigation scenario is framed in the special context of Green Marathon, which is different from the general social behavior or experimental environment in most studies. In an event such as a marathon, individuals are often able to develop strong environmental emotions in the context of perceived group environmental efficacy. This also advances the study of green marathons.

### 5.4 Recommendations for countermeasures

In order to be associated with environmental behaviors more effectively, we suggest strengthening the cultivation of emotionally driven and self-sustainable identity in environmental activities and education. First, research has shown that emotional resonance plays a key role in promoting behavioral change, so environmental activities should focus on storytelling and interactivity with a view to enhancing participants’ emotional experience. For example, in activities such as the Green Marathon, demonstrating the positive impacts of collective action by laying out the emotional stories of environmental action can effectively stimulate environmental empathy in individuals, thus promoting them to view environmental behavior as part of their personal identity. Second, the cultivation of self-sustainable identity is also an important path to achieve long-term environmental behavior. When individuals closely associate environmental behaviors with their own values and lifestyles, they are more likely to transform environmental actions into part of their self-identity. Therefore, organizations and educators can guide individuals to share their environmental stories through social platforms, community activities, and other channels to be associated with their sense of identification with environmental behaviors.

Several boundaries should be noted when interpreting these findings. First, because our data are cross-sectional and self-reported, the analyses do not establish temporal precedence and therefore do not support causal inference. Accordingly, the estimated paths should be interpreted as associations consistent with the proposed CAC-based process, rather than evidence that Green Marathon participation causes changes in empathy, identity, or pro-sustainable behavior. Future studies using longitudinal, cross-lagged, or experimental/event-based designs and incorporating objective behavioral indicators are needed to test directionality and causality more rigorously.

We also consider the extent to which these findings may generalize beyond Green Marathon participants. The proposed CAC-consistent sequence (awareness → empathy → self-sustainable identity → pro-sustainable behavior tendency) may plausibly extend to other green events that combine salient sustainability cues with immersive participation, such as eco-festivals, community clean-up campaigns, or environmental volunteering programs. However, the strength of the empathy-to-identity pathway may depend on event features, including involvement intensity, collective rituals/community belonging, and perceived real-world environmental impact. In lower-involvement or primarily informational settings, affective and identity activation may be weaker, potentially attenuating the sequential mechanism. Future research should compare multiple event types and non-sport sustainability contexts, and test event characteristics as moderators to specify when and for whom the pathway is most likely to operate.

At the same time, in collective environmental activities, the sharing of environmental achievements and the actual results of collective action can be associated with the participants’ collective environmental efficacy and sense of belonging. When individuals see the actual results of collective action, they tend to identify more with their own responsibility as part of the environmental action, and this emotional identification can further be associated with the formation of self-sustainable identity, which in turn is linked to individuals to continue to adopt environmentally friendly behaviors. Therefore, we call for the promotion of more group sustainable activities, such as the University of Melbourne’s charity organization will organize students of different majors and different ages to pick up garbage at the beach. At the same time, we call on the government, sports and environmental organizations, universities and other institutions to give further policy support to be associated with global sustainability.

### 5.5 Theoretical contributions

In advancing the Cognitive-Affective-Conative (CAC) model, this study refines the traditional understanding of the sequence linking cognition, affect, and conation. While CAC has historically been presented as a linear pathway where cognition directly influences behavior through affect and conation, our findings challenge this simplicity, particularly in event-based sustainability contexts. In the case of the Green Marathon, cognition, while crucial, appears to trigger conation primarily after it is first activated through emotional engagement (affect) and later consolidated into a more robust, identity-based motivation. This suggests that conation is not merely a stage that follows cognition in a direct sequence; rather, it is shaped by a deeper, identity-grounded readiness to act.

By introducing the concept of self-sustainable identity, this study provides a psychological layer to the CAC model, showing that behavioral tendencies related to sustainability are not only a function of cognitive recognition but also an outcome of a self-concept that aligns with sustainable actions. This pathway, moving from awareness to empathy, to self-sustainable identity, and ultimately to pro-sustainable behavior tendencies, adds conceptual depth to CAC, emphasizing how sustainability-related behavior can emerge from a more nuanced interplay of cognitive, emotional, and identity factors. Therefore, this research refines the CAC framework by specifying a more complex, layered process that better captures the motivational dynamics at play in real-world sustainability events.

## 6 Conclusion

This study provides a new theoretical framework for understanding how green events be associated with public environmental awareness and behavior by providing an in-depth analysis of how three-dimensional environmental awareness in Green Marathon events is associated with participants’ pro-sustainable behavior tendencies through the chain-mediated effects of environmental empathy and self-sustainable identity. The study shows that (1) environmental value integration, green behavior belongingness, and environmental collective efficacy positively affect pro-sustainable behavior tendency. (2) Environmental empathy and self-sustainable identity play a chain mediating role between environmental value integration and pro-sustainable behavior tendency. (3) Environmental empathy and self-sustainable identity mediated the chain between green behavior belongingness affecting pro-sustainable behavior tendency. (4) Environmental empathy and self-sustainable identity mediated the chain between environmental collective efficacy and pro-sustainable behavior tendency. In summary, green activities such as the Green Marathon should focus on the dissemination of ecological awareness, and moreover should establish more profound and sustainable motivation for environmental behavior through the mechanism of emotional empathy and self-identification. In the future, the promotion strategy of green activities can draw on the findings of this study to further be associated with the overall environmental behavior of the society through the combination of multi-dimensional environmental awareness cultivation and collective action, thus contributing to the realization of the global sustainable development goals.

## 7 Shortcomings and prospects

Although this study employed a three-path chain mediation model to delve into the complex mechanisms linking environmental awareness to pro-sustainable behavior in the context of green marathons, several limitations should be acknowledged. First, the data were collected exclusively in China, which restricts the sample’s diversity and fails to account for potential variations across different ethnic groups, cultural contexts, and levels of economic development. Future research should include participants from multiple countries and diverse demographic backgrounds to be associated with the generalizability of the findings and reduce potential cultural or regional biases.

Second, participants psychological states, such as environmental empathy and self-sustainable identity, may evolve over time and with continued engagement in environmental actions. To better capture these dynamic processes, future studies should adopt longitudinal or cross-lagged panel designs, which would allow for stronger causal inferences regarding how Green Marathon participation is associated with sustained pro-environmental behavior.

Third, the reliance on cross-sectional data limits the ability to establish temporal precedence or causality. Future research could incorporate panel data or leverage objective metrics such as official marathon operational records and carbon footprint assessments to complement self-reported measures. Addressing these limitations in future work will strengthen the validity and practical relevance of the findings.

## Data Availability

The raw data supporting the conclusions of this article will be made available by the authors, without undue reservation.
