# Supplementary material for: How green marathons are associated with pro-sustainable behavior: the chain-mediating roles of environmental empathy and self-sustainable identity
Source: Front Psychol. 2026 Apr 20;17:1731205. doi: 10.3389/fpsyg.2026.1731205 (PMC13135924; doi:10.3389/fpsyg.2026.1731205)
Supplement: Supplementary file 1 [file Supplementary_file_1.DOCX]

Appendix A. The Complete Questionnaire

| **Questionnaire on Green Marathons Promote Pro-Sustainable Behavior** | | | | | | | |
| --- | --- | --- | --- | --- | --- | --- | --- |
| **Category** | **Measurement items** | | **1** | **2** | **3** | **4** | **5** |
| Environmental Value  Integration  (EVI) | EVI1 | I am more concerned about the environmental concepts of the Green Marathon event. | **◎** | **◎** | **◎** | **◎** | **◎** |
|  | EVI2 | I believe the environmental significance of the Green Marathon is more important than its competitive significance. | **◎** | **◎** | **◎** | **◎** | **◎** |
|  | EVI3 | Participating in the Green Marathon makes me pay more attention to environmental behaviors in daily life. | **◎** | **◎** | **◎** | **◎** | **◎** |
|  | EVI4 | The Green Marathon event makes me realize that environmental protection and healthy living are related. | **◎** | **◎** | **◎** | **◎** | **◎** |
|  | EVI5 | I am willing to adjust my participation behavior for the sake of environmental concepts. | **◎** | **◎** | **◎** | **◎** | **◎** |
|  | EVI6 | Environmental concepts were an important reason for my decision to participate in the Green Marathon. | **◎** | **◎** | **◎** | **◎** | **◎** |
| Green Behavior  Belongingness  (GBB) | GBB1 | I feel part of a collective environmental campaign. | **◎** | **◎** | **◎** | **◎** | **◎** |
|  | GBB2 | Practicing environmental protection together with other participants gives me a sense of belonging. | **◎** | **◎** | **◎** | **◎** | **◎** |
|  | GBB3 | In the Green Marathon, I feel we are a team dedicated to environmental protection. | **◎** | **◎** | **◎** | **◎** | **◎** |
|  | GBB4 | When with other environmentally conscious participants, I feel accepted. | **◎** | **◎** | **◎** | **◎** | **◎** |
|  | GBB5 | Participating in the Green Marathon helped me find like-minded people. | **◎** | **◎** | **◎** | **◎** | **◎** |
|  | GBB6 | I am proud to be part of the Green Marathon's environmental efforts. | **◎** | **◎** | **◎** | **◎** | **◎** |
|  | GBB7 | During the event, I connect with others through our shared environmental goals. | **◎** | **◎** | **◎** | **◎** | **◎** |
|  | GBB8 | Participating in collective environmental actions motivates me to persist in environmental behaviors. | **◎** | **◎** | **◎** | **◎** | **◎** |
| Collective  Environmental  Efficacy  (CEE) | CEE1 | Our team always manages to solve difficult environmental problems if we try hard enough. | **◎** | **◎** | **◎** | **◎** | **◎** |
|  | CEE2 | Our team has the ability to make substantial contributions to environmental protection. | **◎** | **◎** | **◎** | **◎** | **◎** |
|  | CEE3 | Even when facing difficulties, our team can find environmental solutions. | **◎** | **◎** | **◎** | **◎** | **◎** |
|  | CEE4 | I believe our team can influence more people to participate in environmental protection. | **◎** | **◎** | **◎** | **◎** | **◎** |
|  | CEE5 | Our team's actions can have a positive impact on the local environment. | **◎** | **◎** | **◎** | **◎** | **◎** |
|  | CEE6 | Compared to other teams, our team is more effective in environmental protection. | **◎** | **◎** | **◎** | **◎** | **◎** |
|  | CEE7 | Our team can overcome resource limitations to practice environmental protection. | **◎** | **◎** | **◎** | **◎** | **◎** |
|  | CEE8 | I believe that through collective effort, we can improve environmental conditions. | **◎** | **◎** | **◎** | **◎** | **◎** |
|  | CEE9 | Our team's environmental actions can inspire others. | **◎** | **◎** | **◎** | **◎** | **◎** |
|  | CEE  10 | Overall, I am confident in our team's ability to solve environmental problems. | **◎** | **◎** | **◎** | **◎** | **◎** |
| Environmental  Empathy  (EE) | EE1 | I am saddened and distressed by the damage done to the environment. | **◎** | **◎** | **◎** | **◎** | **◎** |
|  | EE2 | When I think about natural habitats disappearing, I feel sad. | **◎** | **◎** | **◎** | **◎** | **◎** |
|  | EE3 | Seeing animals suffer due to environmental pollution pains me. | **◎** | **◎** | **◎** | **◎** | **◎** |
|  | EE4 | I can feel the "pain" of the natural world. | **◎** | **◎** | **◎** | **◎** | **◎** |
|  | EE5 | Seeing others' environmentally destructive behavior makes me angry. | **◎** | **◎** | **◎** | **◎** | **◎** |
|  | EE6 | I care about environmental issues as much as I care about important people in my life. | **◎** | **◎** | **◎** | **◎** | **◎** |
|  | EE7 | Hearing news about environmental degradation makes me feel worried. | **◎** | **◎** | **◎** | **◎** | **◎** |
|  | EE8 | I feel sympathy for living beings affected by environmental problems. | **◎** | **◎** | **◎** | **◎** | **◎** |
| Self-Sustainability  Identification  (SSI) | SSI1 | I am aware of my role and responsibility in promoting sustainability. | **◎** | **◎** | **◎** | **◎** | **◎** |
|  | SSI2 | Practicing a sustainable lifestyle strengthens my sense of self-identity. | **◎** | **◎** | **◎** | **◎** | **◎** |
|  | SSI3 | Promoting sustainable development is an important part of my personal identity. | **◎** | **◎** | **◎** | **◎** | **◎** |
|  | SSI4 | I want others to see me as "someone who advocates for sustainability". | **◎** | **◎** | **◎** | **◎** | **◎** |
|  | SSI5 | Participating in the Green Marathon reinforces my self-perception as a sustainability practitioner. | **◎** | **◎** | **◎** | **◎** | **◎** |
| Pro-Sustainable  Behavior Tendency (PSBT) | PSBT | I will continue to adopt a sustainable lifestyle after the Green Marathon. | **◎** | **◎** | **◎** | **◎** | **◎** |
|  | PSBT | I am willing to choose more environmentally friendly products in my daily life. | **◎** | **◎** | **◎** | **◎** | **◎** |
|  | PSBT | I will try to influence people around me to practice sustainable behaviors. | **◎** | **◎** | **◎** | **◎** | **◎** |
|  | PSBT | I will continue to participate in similar environmental initiatives in the future. | **◎** | **◎** | **◎** | **◎** | **◎** |
|  | PSBT | Overall, I am willing to make more efforts for sustainable development. | **◎** | **◎** | **◎** | **◎** | **◎** |
| (1: strongly disagree, 2: disagree, 3: neither agree nor disagree, 4: agree and 5: strongly agree) | | | | | | | |
